# DNA Damage Response

**DOI:** 10.3390/biom11010123

**Published:** 2021-01-19

**Authors:** Valentyn Oksenych, Denis E. Kainov

**Affiliations:** 1 Department for Cancer Research and Molecular Medicine (IKOM), Norwegian University of Science and Technology, 7491 Trondheim, Norway; 2 Department of Biosciences and Nutrition (BioNuT), Karolinska Institutet, 14183 Huddinge, Sweden; 3 KG Jebsen Centre for B Cell Malignancies, Institute of Clinical Medicine, University of Oslo, 0316 Oslo, Norway; 4 Institute of Clinical Medicine, University of Oslo, 0318 Oslo, Norway; 5 Institute of Technology, University of Tartu, 50090 Tartu, Estonia

DNA in our cells is constantly modified by internal and external factors. For example, metabolic byproducts, ionizing radiation (IR), ultraviolet (UV) light, and medicines can induce spontaneous DNA lesions [1,2,3]. However, DNA modifications can also be programmed. In particular, the recombination activating gene (RAG) can induce breaks generated during the V(D)J recombination in developing B and T lymphocytes [1,2]. In addition, activation-induced cytidine deaminase (AID) makes DNA break during the class-switch recombination (CSR) and somatic hypermutation (SHM) in B cells [1,2].

One focus of this Special Issue is on the non-homologous end-joining (NHEJ) DNA repair pathway and DNA repair and DNA damage response (DDR) factors. Oksenych et al. and others found the functional redundancy of these factors in mammalian cells. In particular, a genetic interaction was found between the X-ray repair cross-complementing protein 4 (XRCC4)-like factor (XLF, also known as Cernunnos) and the DNA-dependent protein kinase catalytic subunit (DNA-PKcs) [4,5], the paralog of XRCC4 and XLF (PAXX) [6,7,8,9], and the modulator of retrovirus infection (MRI, also known as Cyren) [10]. Moreover, a genetic interaction was found between the NHEJ factor XLF and DDR factors, including Ataxia telangiectasia mutated (ATM) [11], histone H2AX [11], and p53-binding protein 1 (53BP1) [12].

Another aspect of this Special Issue is the various anti-cancer therapies and their combinations. These therapies, such as IR and cisplatin, induce extensive DNA damage in rapidly growing cells [3]. These DNA damages, if unresolved over time, can induce DDR and cell cycle checkpoint arrest, which leads to p53-mediated Bcl-xL-controlled apoptosis [13].

In addition, Castaneda-Zegarra et al. [14] describe the generation and characterization of a mouse model lacking the NHEJ factor MRI. The MRI-deficient mice exhibited a nearly normal development and life-span. The MRI-deficient mice did not show any detectable alterations in the count of mature B and T lymphocytes when compared to heterozygous and wild-type (WT) controls. The development of the brain in these mice was also normal. One detectable phenotype was a significant reduction in CSR levels in MRI-deficient mice when compared to WT controls.

Furthermore, Beck et al. [15] presented a double-deficient model lacking XLF and a DDR factor, the mediator of DNA damage checkpoint protein 1 (MDC1). While single-deficient *Xlf*^−/−^ and *Mdc1*^−/−^ mice were alive, the double-deficient *Xlf^−/−^Mdc1*^−/−^ mice were embryonic lethal. Progenitor B cell lines (vAbl) lacking both XLF and MDC1 possessed significantly reduced levels of V(D)J recombination when compared to single-deficient and WT controls.

Translesion DNA synthesis (TLS) is a major source of the point mutations accumulating in the genomes of our cells. In addition, mammalian DNA-dependent RNA polymerases are also able to introduce mistakes into the RNA structure and, thus, result in modified proteins via “transcriptional mutagenesis”. Rodriguez-Alvarez et al. [16] developed and presented a new technology in order to detect TLS. In particular, the authors present the enhanced green fluorescent protein (EGFP)-based reporter that can be used for the direct and sensitive detection of mutagenic DNA lesions bypasses.

Seelinger et al. [17] generated and characterized single-knockout human cell lines lacking helicase-like transcription factor (HLTF), SNF2, histone-linker, PHD, and RING finger domain-containing helicase (SHPRH), and a double-knockout HLTF/SHPRH. Various DNA damage types were introduced in these cells using UV light, methyl methanesulfonate (MMS), mitomycin C (MMC), and cisplatin. The authors identified both common and distinct functions of HLTF and SHPRH in human cells and suggested a model with SHPRH being a central player in regulating DDR via a protein kinase CHK2.

Finally, Naumenko et al. [18], in an elegant in vitro-based study, demonstrated that Y-box-binding protein 1 (YB-1) likely regulates the cellular events of PARylation via poly(ADP-ribose) (PAR) and DNA.

Future research in DNA repair and DDR will focus on identifying new factors facilitating these processes, elucidating the specific functions of these factors and their mechanisms of action. Such research will be challenged by the complex genetic interactions between DDR factors. Research in this area will benefit from introducing novel technologies and model systems. It will facilitate the development and approval of medicines and their combinations for the treatment of various cancers, immune disorders, and viral infections.
